# Six years' experience of in-house maintenance of clinical chemistry autoanalysers

**DOI:** 10.1155/S1463924683000498

**Published:** 1983

**Authors:** D. J. Wyper, G. A Corner, L. B. Roberts, A. C. A. Glen, A. MeLelland, E. Simpson

**Affiliations:** 1 West of Scotland Health Boards Department of Clinical Physics and Bio-Engineering 11 West Graham Street Glasgow G4 9LF UK; 2 Department of Biochemistry Gartnavel General Hospital Glasgow G12 0HX UK; 3 Department of Biochemistry Victoria Infirmary Glasgow G42 9TY UK; 4 Department of Biochemistry Royal Infirmary Glasgow G4 0SF UK; 5 Department of Biochemistry Monklands District General Hospital Monkscourt Avenue Airdrie UK


Journal of Automatic Chemistry, Volume 5, Number 4 (October–December 1983), pages 201–203

### Six years' experience of in-house maintenance of clinical chemistry autoanalysers

**D. J. Wyper, G. A Corner**

*West of Scotland Health Boards, Department of Clinical Physics and Bio-Engineering, 11 West Graham Street, Glasgow G4 9LF, UK*

**L. B. Roberts**

*Department of Biochemistry, Gartnavel General Hospital, Glasgow G12 0HX, UK*

**A. C. A. Glen**

*Department of Biochemistry, Victoria Infirmary, Glasgow G42 9TY, UK*

**A. McLelland**

*Department of Biochemistry, Royal Infirmary, Glasgow G4 0SF, UK*

**and E. Simpson**

*Department of Biochemistry, Monklands District General Hospital, Monkscourt Avenue, Airdrie, UK*

### Introduction

In April 1976 a scheme for in-house maintenance of clinical chemistry autoanalysers in the West of Scotland region was started. Administration of the scheme was undertaken by the Greater Glasgow Health Board with cross-accounting to the five other Area Health Boards in the West of Scotland. Details of this were presented in an earlier publication [1]. The purpose of this article is to describe the progress of the service over a six-year period, to present experience in taking new items of equipment into the scheme and to explain how some cost-effective development work was undertaken.

The objective of the in-house maintenance scheme is to provide a service within the National Health Service (NHS) which produces cost savings, and which provides equipment maintenance of an acceptably high standard. The remit is therefore:

(1) To provide a cost-effective service.
(2) To maintain the equipment to a specified standard.
(3) To sustain this quality of service in the short term (i.e. holidays and sickness leave) and in the long term (i.e. staff turnover).

Factors to consider in assessing in-house maintenance are:

(4) How it affects the relationship between the equipment user and the manufacturer or supplier, and how it affects the ability of the manufacturer to sustain a viable maintenance division of his own.
(5) Whether it benefits other companies by subcontracting or purchasing.
(6) Whether there are secondary benefits for the Health Board.

### Cost effectiveness

As shown in table I, the cost savings to the West of Scotland Health Boards have been substantial—averaged over the six-year period the savings amount to £83 000 per annum. The cost of running the service was £57 000 per annum, compared with a commercial equivalent of £140 000 per annum.

Details of the costing method have not changed significantly since the previous publication [1]. Expenditure includes remuneration plus a 50% overhead to cover employer's National Insurance and Superannuation contributions, clerical and administrative assistance, rates, fuel and building maintenance costs. Other items charged out are recruitment, training-course fees, purchase of tools and test equipment, purchase of vehicles and travelling expenses, purchase of manuals and other sundry expenses. These fixed costs are charged out to each District or Area on a *pro rata* basis depending upon the amount of equipment within that Area. Parts used are charged out retrospectively to each Area or District.

### Standard of maintenance

Although the day-to-day contact between the maintenance team and the autoanalysers' operators and heads of clinical laboratories ensured that there were no major shortcomings in the

*Table 1. Costs and savings of in-house maintenance.*

| Year | Cost of in-house scheme | Cost of commercial equivalent | Saving |
|---|---|---|---|
| 1976/1977 | £50 000 | £110 000 | £60 000 |
| 1977/1978 | £35 000 | £110 000 | £75 000 |
| 1978/1979 | £48 000 | £130 000 | £82 000 |
| 1979/1980 | £57 000 | £151 000 | £94 000 |
| 1980/1981 | £74 000 | £161 000 | £87 000 |
| 1981/1982 | £79 000 | £178 000 | £99 000 |

*Notes*
(1) The first year is anomalous. The in-house costs were high due to initial stocking of spare parts and loan modules, and the commercial equivalent includes a £35 000 charge to 'update' systems prior to acceptance on a maintenance scheme.
(2) These savings make no allowance for indirect savings, such as the extension of the functional life of equipment.

service, a questionnaire was devised and issued in order to reappraise the effectiveness of different aspects of the service. This was done in February 1979 and referred only to continuous-flow autoanalysers, which at that time constituted the bulk of the equipment for which the team was responsible.

Users were asked to rate the service on a scale ranging from '1' (unsatisfactory) to '10' (excellent) for planned preventive maintenance and for response to breakdowns. The results are shown in table 2. Although no data exists with which to compare these findings, they are reassuring. With the exception of one user who clearly had a problem following his annual service, all ratings are above average. As would be expected of a local in-house service, it rates extremely highly in response time to breakdowns. The knowledge that the maintenance team can, in most instances, arrive within an hour of a fault being reported is extremely reassuring where an acute analytical service is being provided. The technical expertise of the in-house staff is obviously satisfactory.

This latter point is important as one fear which companies had expressed about in-house maintenance was that the equipment might get a bad name and that sales would suffer accordingly. It has been demonstrated, however, that a high-calibre service engineer can be attracted to an in-house maintenance scheme.

*Table 2. Questionnaire completed by laboratories using in-house maintenance.*

*Planned Preventive Maintenance (PPM).*

(1) Rate the down time during PPM visits:

| | 1 | 2 | 3 | 4 | 5 | 6 | 7 | 8 | 9 | 10 | |
|---|---|---|---|---|---|---|---|---|---|---|---|
| Unsatisfactory | - | - | - | - | - | 1 | - | 18 | 6 | 1 | Excellent |

(2) Rate the analytical quality-control following PPM:

| | | | | | | | | | | | |
|---|---|---|---|---|---|---|---|---|---|---|---|
| Unsatisfactory | - | - | - | - | - | 14 | 1 | 5 | 6 | - | Excellent |

(3) Rate the effectiveness of PPM in eliminating breakdown due to wear and tear:

| | | | | | | | | | | | |
|---|---|---|---|---|---|---|---|---|---|---|---|
| Unsatisfactory | - | 1 | - | - | - | 5 | 9 | 7 | 4 | - | Excellent |

*Breakdowns*

(4) Rate the speed of response to tackling a problem:

| | | | | | | | | | | | |
|---|---|---|---|---|---|---|---|---|---|---|---|
| Unsatisfactory | - | - | - | - | - | 2 | 2 | 1 | 4 | 17 | Excellent |

(5) Rate the ability to get front-end equipment operational:

| | | | | | | | | | | | |
|---|---|---|---|---|---|---|---|---|---|---|---|
| Unsatisfactory | - | - | - | - | - | - | - | 7 | 7 | 12 | Excellent |

(6) Rate the ability to get back-end equipment operational:

| | | | | | | | | | | | |
|---|---|---|---|---|---|---|---|---|---|---|---|
| Unsatisfactory | - | - | - | - | - | 1 | - | 5 | 6 | 12 | Excellent |

### The stability of in-house maintenance

The most important factors governing the viability of an in-house scheme from the staffing point of view are, firstly, optimizing the size of the team; and, secondly, retaining members of staff in post for a sufficiently long period of time.

Staffing levels progressed as shown in table 3. Increases in the staff numbers generally corresponded to increased work-load, and were agreed by the Health Boards on the basis of cost effectiveness. The most significant expansion took place when some centrifugal analysers were taken into the scheme.

One of the attractions of a medical physics department to staff is that they can have careers which cover a variety of disciplines. This is a healthy situation and one which is to be encouraged. A mean job occupancy varying from two years for a Medical Physics Technician (MPT) Grade III to four years for an MPT Grade I or Principal Physicist is desirable. In this way the more senior staff can ensure a continuity of expertise within the section and the junior staff, although initially learning, stay for a sufficient length of time to contribute much useful work. The mean occupancy figures attained are 25 months for MPT Grade IIIs and 40 months for the remainder of the staff. With one exception all members of staff moved to other appointments within the Department of Clinical Physics and Bio-Engineering.

*Table 3. Staffing.*

| Year | Principal Physicist | MPT Grade I | MPT Grade II | MPT Grade III |
|---|---|---|---|---|
| 1976/1977 | 1 | | 2 | |
| 1977/1978 | 1 | | 2 | |
| 1978/1979 | 1 | | 2 | 1 |
| 1979/1980 | 1 | | 3 | 1 |
| 1980/1981 | 1 | 1* | 2* | 1 |
| 1981/1982 | 1 | 1 | 2 | 1 |

* One MPT Grade II was upgraded to MPT Grade I in December 1981.

*Note*
MPT (Medical Physics Technician) postings range from Principal, though Grades I to IV.

### Relationship between the user and the equipment supplier/manufacturer

In terms of the general attitude of companies to in-house maintenance, most have been generally in favour of some degree of customer involvement. In this particular case it may be influenced by the relative remoteness of the West of Scotland region from the south of England. The service is more acceptable to companies who do not have an existing maintenance establishment based in the West of Scotland.

Regular dialogue between laboratory personnel and equipment manufacturers is vital for the development and marketing of improved autoanalysers. In an attempt to ensure that users do not become remote from a manufacturer's latest developments, and that the equipment manufacturers do not lose touch with the thoughts and requirements of users, a Centrifugal Analyser Users' Group was set up. This group meets about four times per year; it is attended by manufacturers' representatives and provides a forum for early presentation of new work from the manufacturer or from laboratory personnel.

### Distributed purchasing

One factor which is pertinent to in-house maintenance and which does not find particular favour with analyser manufacturers is that their monopoly over supplies of spares can be broken. Figure 1 shows details of purchasing spares in 1981–1982.

The bulk of the expenditure in Categories 1 and 2 of figure 1 is for specialized mechanical parts used in planned preventive maintenance. Most of the electronic components listed under Category 3 were readily obtained from the larger electronic component distributors.

Most specialized parts for centrifugal analysers are obtainable only from the manufacturer, but experience so far has been that the need to replace expensive parts has not been great and that the prices have been reasonable.

In principle, the greater distribution of purchasing must lead to an opening up of the market, more competitive pricing and quality assurance.

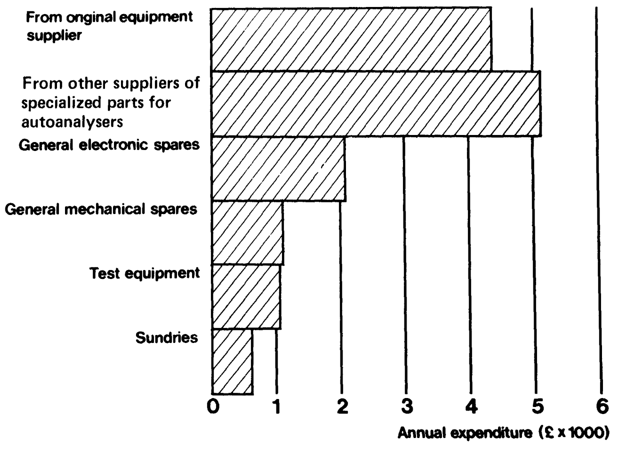

*Figure 1. Categories of spending.*

### Secondary benefits

The most significant secondary benefits associated with an in-house scheme are as follows:

*Help with problems outside the usual remit*
On numerous occasions simple front-line maintenance on equipment outside the scheme's remit has been undertaken. This has normally amounted to a brief appraisal of the instrument concerned, lasting no more than 30 min and involving a discussion of the problem with the operator, a visual inspection of mechanical and electronic parts of the instrument and simple electrical checks on power-supply lines.

Equipment modification and computer interfacing are areas where more time is required and where it is necessary to be more selective in the tasks undertaken. Two examples of work in this area are listed below.

*Extension of the life of existing equipment*
A seven-year amortization period for clinical chemistry equipment is generally accepted by Health Boards as reasonable. Table 4 shows the age distribution of the equipment serviced in 1982–1983 on a routine basis. Since 45% of the equipment is over seven years old (this does not include upgraded equipment which is classed as being only a year old) an extension of the useful working life is being achieved.

*Table 4. Age of equipment serviced in 1982–1983.*

| Age range | Percentage* |
|---|---|
| 1 | 26·2 |
| 2–7 | 28·5 |
| 7–10 | 18·6 |
| >10 | 26·7 |

* Based on fixed costs value for maintenance.

*Using experience to do cost-effective development work*
It would be surprising if involvement in equipment maintenance did not lead to some development ideas, either for production of interfacing modules or for replacing some modules with modules of the team's own design.

Computer interface units for centrifugal and discrete autoanalysers and control modules for continuous-flow autoanalysers have been successfully produced. This development work would not have been possible if the team had not been part of a large medical physics department with good mechanical, electronic and microprocessor development facilities.

Some of the projects were of considerable financial benefit to the West of Scotland Health Boards. The centrifugal analyser interface units were developed in house and then 'sold' to the analyser's manufacturer on a parts-exchange basis. A continuous-flow autoanalyser control unit (ARCAMS) was developed with commercial backing from a British company who are now marketing it. Technical details of this will appear in a subsequent article. Six autoanalysers in the West of Scotland and several in the rest of the UK are now running routinely with ARCAMS control units. This unit should extend the life of these analysers by one generation, as the front-end modules were given a complete overhaul on installation of the control unit. The cost benefits to the West of Scotland Health Boards arising from this work amount to the purchase price of six electrolyte analysers (four six-channel and two 10-channel instruments), less the cost of the upgrade (£14 000 per instrument on average) plus subsequent savings on maintenance.

These cost benefits are not included in the data presented earlier in table 1, which refers only to direct maintenance.

### Conclusion

It has been demonstrated that in-house maintenance can work effectively within the NHS, producing financial savings and improving the technical knowledge within the Health Boards. This is, however, not necessarily a universal conclusion and account must be taken of the volume of work and the geographical distribution of the laboratories involved.

### Reference

1. Wyper, D. J., Porter, D., Etchells, A. H., Fleck, A., Kenny, A. P. and McLelland, A. S. *Annals of Clinical Biochemistry*, **16** (1979), 240.

THIRD EUROPEAN SYMPOSIUM ON THERMAL ANALYSIS AND CALORIMETRY

*9–15 September 1984 in Switzerland*

ESTAC 3 is being organized by members of the European Societies for Thermal Analysis, Calorimetry and Chemical Thermodynamics; it is to be held at the Congress-Center-Casino Interlaken (CCCI). Sessions on the following subjects are being arranged:

Inorganic chemistry/metallurgy
Earth sciences
Organic chemistry/polymers
Biological sciences including medicine and pharmacy
Industrial applications
Theory and instrumentation.

*Further details from ESTAC 3 84, Dr E. Marti, Im Langen Loh 181, CH 4054 Basel, Switzerland.*

